# Treatment and Prognosis of Anaplastic Thyroid Carcinoma: Experience from a Single Institution in China

**DOI:** 10.1371/journal.pone.0080011

**Published:** 2013-11-05

**Authors:** Chuanzheng Sun, Qiuli Li, Zedong Hu, Jiehua He, Chao Li, Guojun Li, Xiaofeng Tao, Ankui Yang

**Affiliations:** 1 Department of Head and Neck Surgery, the Third Affiliated Hospital of Kunming Medical University, Kunming, Yunnan, People’s Republic of China; 2 Department of Head and Neck Surgery, Sun Yat-sen University Cancer Center, Guangzhou, Guangdong, People’s Republic of China; 3 State Key Laboratory of Oncology in South China, Guangzhou, Guangdong, People’s Republic of China; 4 Department of Pathology, Sun Yat-sen University Cancer Center, Guangzhou, Guangdong, China; 5 Department of Head and Neck Surgery, The Tumor Hospital of Sichuan Province, Chengdu, Sichuan, People’s Republic of China; 6 Department of Head and Neck Surgery, The University of Texas MD Anderson Cancer Center, Houston, Texas, United States of America; 7 Radiology Department of Shanghai Ninth People’s Hospital Affiliated Shanghai JiaoTong University School of Medicine, Shanghai, People’s Republic of China; Consiglio Nazionale delle Ricerche (CNR), Italy

## Abstract

**Background:**

Anaplastic thyroid carcinoma (ATC), a highly aggressive malignancy, has a poor prognosis, and the consensus on the most effective treatment is needed.

**Methods:**

Clinical data from all ATC patients treated in our institution over a 30-year period (between May 1980 and May 2010) were analyzed retrospectively with regard to mortality and survival rates (Kaplan–Meier). Multivariate analysis was performed using a Cox proportional hazards model.

**Results:**

Sixty cases were analyzed. The overall 1- and 3-year survival rates were 35.0% and 22.9%, respectively. Univariate analysis showed that the best prognosis was seen in patients younger than 55 years, those without distant metastases, those with white blood cell (WBC) counts < 10.0 × 10^9^/L or blood platelet (PLT) counts < 300.0 × 10^9^/L at presentation, those who did not receive chemotherapy, and those who received radiotherapy doses ≥ 40 Gy or underwent surgery plus postoperative radiotherapy. According to multivariate analysis, the WBC count at first presentation and the type of therapeutic regimen independently influenced survival.

**Conclusions:**

We found that the elevated peripheral PLT count may be an adverse prognostic factor of ATC patients. The prognosis for ATC is especially poor for patients with distant metastasis, a WBC count ≥ 10.0×10^9^/L, a PLT count ≥ 300.0 × 10^9^/L, or age ≥ 55 years. WBC count at presentation and surgery with or without postoperative radiotherapy independently influenced the prognosis. Intensive treatment combining surgery with postoperative radiotherapy is recommended for ATC patients with stage IVA/B disease.

## Introduction

Anaplastic thyroid carcinoma (ATC) accounts for 1.6%–5% of all thyroid cancers and is one of the most aggressive malignant tumors in humans. Only 20% of affected patients survive for 1 year after diagnosis, and the median survival duration is 3–9 months. Additionally, ATC is responsible for 14%–40% of thyroid carcinoma-related deaths [1–8]. ATC patients are typically elderly, with most older than 60 years, and manifests as a rapidly enlarging anterior neck mass that is accompanied by dyspnea, dysphagia, and vocal cord paralysis. Furthermore, 20%–50% of patients present with distant metastases, most often pulmonary [1–3,6,9]. Multimodal therapy can achieve better results in terms of avoiding death from local invasion and suffocation[1,10], but no firm conclusions have been reached concerning treatment regimens[7-14].Thus, there is a need to investigate the effects of multimodal therapy, to determine prognostic factors in ATC, and to provide practical and constructive information for clinicians. In this study, we conducted a retrospective review of a single institution’s experience with ATC over 3 decades and examined the clinical features, treatment efficacy, and prognostic factors that affected patient survival.

## Materials and Methods

### Clinical Data

The study was approved by the Ethic Committee of the Sun Yat-sen University Cancer Center; and written informed consent was obtained from all study subjects prior to enrollment. The selection criterion was that all consecutive patients pathologically diagnosed with ATC in Sun Yat-sen University Cancer Center between May 1980 and May 2010 were enrolled. All of the pathological slides were reviewed again before this study by a single expert pathologist. ATC diagnosis was established on the basis of histologic or cytologic features and confirmed by immunohistochemical staining when necessary to ensure the exclusion of poorly differentiated thyroid carcinoma or lymphoma. Data were extracted for all patients with a finally confirmed diagnosis of ATC, including age and sex of the patient; dates of diagnosis, start of treatment, and last follow-up; methods of diagnosis; white blood cell (WBC) count; blood platelet (PLT) count; tumor size; distant metastasis; acute symptoms (dysphonia, dysphagia, dyspnea); rapid growth of the tumor; methods of treatment; outcome and cause of death; T status, N status and stage according to the tumor-node-metastasis classification of the American Joint Committee on Cancer (AJCC) [15]. At the time of diagnosis, the tumor size and neck nodal status were determined by clinical and radiologic examinations, including computed tomography (CT), magnetic resonance imaging, and ultrasonography. To diagnose distant metastasis, the patients’ physicians used chest radiography, ultrasonography, bone scintigraphy, CT, and/or positron emission tomography/CT.

### Immunohistochemical staining of erythropoietin (EPO), interleukin-6 (IL-6), IL-11, colony-stimulating factor 1 (CSF1)

The paraffin-embedded ATC tissue specimens were cut into 4 μm sections and baked at 60 °C for 2 hours. All sections were deparaffinized with xylenes and rehydrated with graded ethanol to distilled water. After being treated with 3% H_2_O_2_ for 30 min to block the endogenous peroxidase, sections were submerged in EDTA antigen retrieval buffer (pH 8.0) and microwaved for antigen retrieval. Then the sections were treated with normal goat serum for 30 min to reduce the nonspecific binding and incubated with rabbit anti-EPO and mouse anti-IL-6 (Santa Cruz Biotechnology, Santa Cruz, CA,USA), rabbit anti-M-CSF (Eptomics, Abcam, USA) or rabbit anti-IL-11 (NOVUS Biologicals, novusbio, USA ) for overnight at 4 °C (dilutions of: anti-EPO 1:50, anti-IL-6 1:100, anti-M-CSF 1:200, anti-IL-11 1:250).The slides were washed with PBS (the proportion of tween-20 is 1:1000) three times and incubated with the anti-rabbit or anti-mouse secondary antibody for 30 min at 37 °C. For color reaction, diaminobenzidine (DAB, Zhongshan biological and technical company, Beijing, China) was used. For negative controls, the antibody was replaced by normal goat serum. The expression levels were determined by scoring the percentage and intensity of the positively stained tumor cells. All slides were assessed independently by a pathologist who did not know the identity of the patient and clinical outcome.

### Follow-Up and Statistical Analysis

Follow-up information was collected by letter, telephone, which was conducted regularly by the Department of Follow-up, Sun Yat-sen University Cancer Center, or by face-to-face outpatient interviews. Overall survival (OS) duration was calculated from the date of the definitive diagnosis until the day of death or the last date in the medical records on which the patient was reported to be alive. All of the data were analyzed using SPSS 18.0 software (SPSS, Chicago, IL). Survival curves were generated using the Kaplan–Meier method, and the log-rank test was used for univariate analysis. For multivariate survival analysis, a Cox proportional hazards model was used, and *P* < 0.05 was considered statistically significant.

## Results

### Patient Characteristics

Over the past 30 years, 60 patients with ATC were referred to our institution (Table 1). The age at first diagnosis ranged from 27 to 80 years, with a median age of 58 years. The patients with ATC were clinically classified into four types according to the standard described by Sugitani [5]: common type (n = 47); incidental type (n = 5); anaplastic transformation at the neck lymph node(s) (n = 8); and no anaplastic transformation at a distant site. At the time of diagnosis, 44 (73.3%) patients presented with a sudden increase in the size of a thyroid mass. Upper airway obstruction caused by extrinsic compression, vocal cord paralysis, tumor invasion of the trachea, or trachea ingrowth was observed in 34 (56.7%) patients. Twenty-three (38.3%) patients presented with signs of hoarseness, 14 (23.3%) presented with dyspnea, and 6 (10.0%) presented with dysphagia. Distant metastasis occurred in 16 (26.7%) (bilateral lung metastases in 9 patients, bilateral lung and bone in 1, bone in 3, and unilateral lung in 3). 

**Table 1:** Survival analysis according to clinical features and type of therapy in patients with ATC.

Prognostic factors	Univariate analysis					Multivariate analysis		
	Median Survival (months)	1-year OS (%)	3-year OS (%)	*P*		HR	95% CI	*P*
Sex								
Men (n = 28)	5	32.1	21.4	0.684				
Women (n = 32)	8	37.5	24.3			0.879	0.469-1.646	0.686
Age								
< 55 years (n = 26)	15	53.8	38.5	0.033				
≥ 55 years (n = 34)	6	20.6	11.0			1.829	0.984-3.397	0.056
Primary tumor size								
< 6 cm (n = 31)	8	29.0	15.1	0.396				
≥ 6 cm (n = 29)	11	41.4	31.0			1.075	0.566-2.044	0.825
Lymph node metastasis								
N0 (n = 24)	8	37.5	28.6	0.230				
N1 (n = 36)	6	33.3	19.4			1.206	0.619-2.349	0.582
Distant metastasis								
M0 (n = 44)	8	38.6	29.1	0.032				
M1 (n = 16)	3	25.0	6.3			0.607	0.129-2.845	0.526
Stage (AJCC, 2010)								
IVA (n = 4)	86	100.0	75.0	0.030				
IVB (n = 40)	8	32.5	24.5			1.798	0.486-6.655	0.379
IVC (n = 16)	3	25.0	6.3					
WBC count								
< 10.0 × 10^9^/L (n = 38)	11	44.7	33.8	0.006				
≥ 10.0 × 10^9^/L (n = 22)	3	18.2	4.5			2.105	0.962-4.605	0.062
*PLT count								
< 300.0 × 10^9^/L (n = 30)	8	33.3	25.9	0.025				
≥ 300.0 × 10^9^/L (n = 12)	3	16.7	0.00					
Surgery type								
None (n = 19)	6	31.6	15.8	0.998				
Thyroidectomy alone (n = 25)	7	32.0	27.4			1.219	0.823-1.804	0.323
Thyroidectomy plus neck dissection (n = 16)	11	43.8	25.0					
Chemotherapy								
Yes (n = 20)	6	20.0	10.0	0.046				
No (n = 40)	9	42.5	29.5			1.310	0.689-2.488	0.410
Radiotherapy								
None or dose < 40 Gy (n = 37)	5	24.3	15.4	0.014				
Dose ≥ 40 Gy (n = 23)	14	52.2	34.8			1.140	0.506-2.566	0.752
Therapeutic regimen								
Surgery plus postoperative radiotherapy (n = 15)	27	73.3	46.7	0.002				
Other therapy (n = 45)	5	22.2	15.0			0.448	0.190-1.058	0.067

Abbreviations: ATC, anaplastic thyroid carcinoma; OS, overall survival; HR, hazard ratio; CI, confidence interval; AJCC, American Joint Committee on Cancer; WBC, white blood cell; PLT, blood platelet.

*Because 18 patients did not receive PLT count examination at presentation, so only the other 42 cases were used to analyze the relationship between PLT count and prognosis.

### Therapeutic Regimen

The 60 patients underwent various treatment regimens. Two patients refused any surgery or chemoradiotherapy because of advanced disease stage or the presence of other medical problems. Chemotherapy alone was administered to 6 patients, and radiotherapy alone was administered to 6 patients. Surgical resection alone was performed in 12 patients. Only tracheostomy and/or biopsy weren’t regarded as surgical treatment in the present study (n = 5). Twenty-nine patients received multimodal treatments: surgery with postoperative radiotherapy (15 patients), surgery with postoperative chemoradiotherapy (8 patients), and surgery with chemotherapy (6 patients).

Surgical removal of the thyroid and cervical nodes, when possible and necessary, was generally the first step in treatment. Two operative procedures were performed: thyroidectomy alone (total, near-total, or subtotal thyroidectomy; n = 25) and thyroidectomy combined with neck dissection (radical neck dissection in 5 cases, modified neck dissection in 8 cases, and selective neck dissection in 3 cases; n = 16). Data on tumor resection margins were unavailable for one third of the patients who underwent surgery; therefore, it was difficult to determine whether these operative procedures should be classified as R0 (no residual tumor), R1 (microscopic residual tumor), or R2 (macroscopic residual tumor).

Twenty-three patients received external beam radiotherapy at doses ≥ 40 Gy (range: 44–78 Gy); 6 patients received external beam radiotherapy at doses of < 40 Gy (range: 14–38 Gy), with a 2-Gy dose administered at each session (5 per week). Twenty patients were treated with chemotherapy. The chemotherapy regimens included doxorubicin and cisplatin in 4 cases; bleomycin, 5-fluorouracil, and cisplatin in 9 cases; and paclitaxel and cisplatin in 7 cases. The median duration of chemotherapy was 3 cycles, ranging from 2 to 4 cycles.

### Follow-Up

The median follow-up was 8 months (range: 2–237 months). Three patients died during treatment: one died from grade IV bone-marrow toxicity caused by chemotherapy, one from pneumonia caused by bilateral vocal cord paralysis, and one from massive hemorrhage caused by a tumor penetrating the common carotid artery wall. Fifty-three patients died during follow-up. One patient died from a nontumor cause, 18 from distant metastasis (found after treatment in 2 patients), and 34 from locoregional disease progression. The remaining 4 living patients were followed until May 2012. Thus, no patients were lost for follow-up in this study. The OS rates at 1 and 3 years for all 60 patients were 35.0% and 22.9%, respectively. No significant difference in OS for the patients with the common type, the incidental type, or the type of anaplastic transformation at the neck was found, their median survival were 6, 7, and 8 months, respectively (*P* = 0.559). 

### Prognosis

#### Univariate analysis

**Figure 1:**
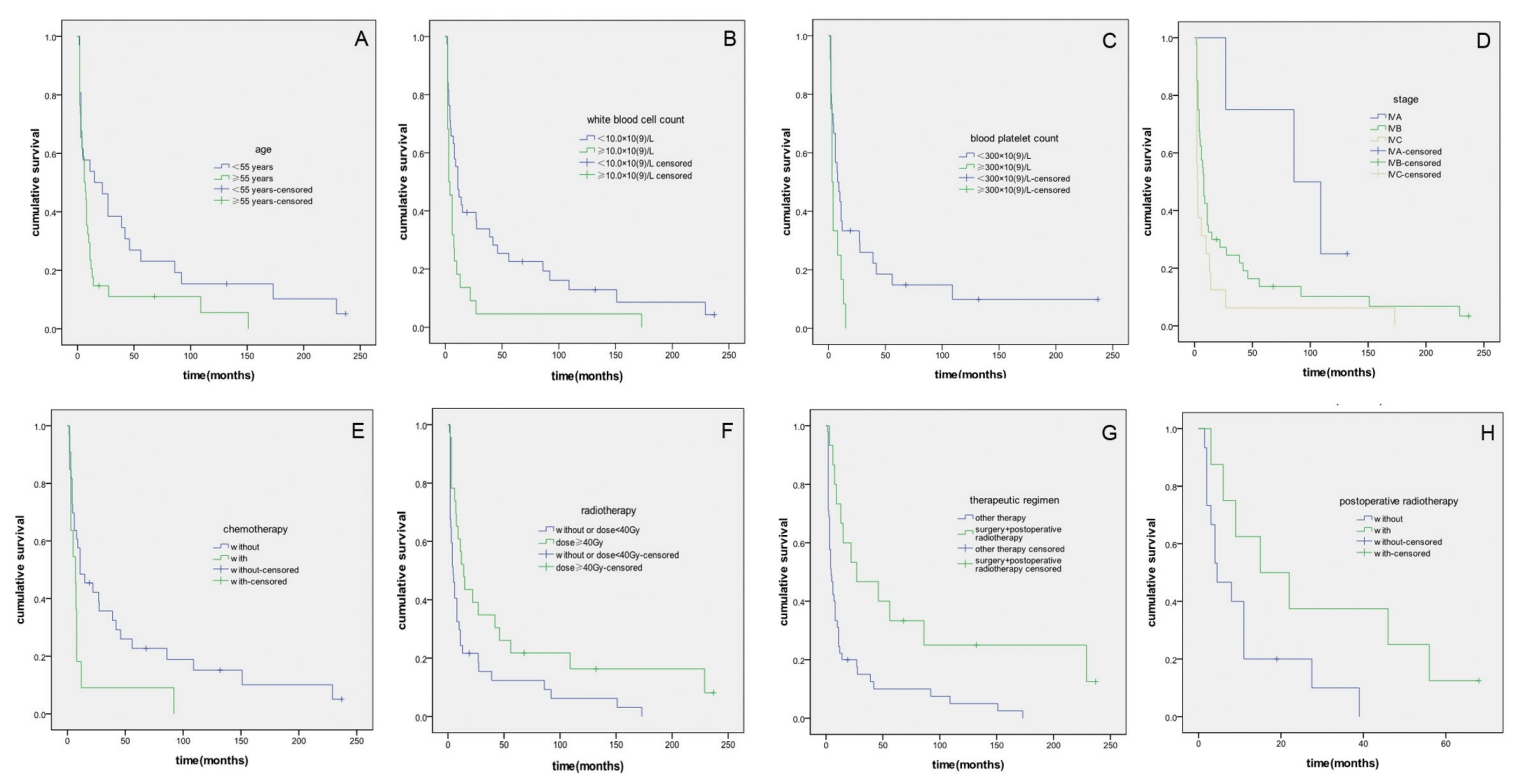
Survival curves for patients with ATC. (A) Survival curves for patients with different ages (*P* = 0.033). (B) Survival curves for patients with different WBC counts (*P* = 0.006). (C) Survival curves for patients with different PLT counts (*P* = 0.025); this analysis contained 42 patients whose PLT counts were measured at their initial presentations. (D) Survival curves according to clinical tumor-node-metastasis stage (*P* = 0.030). (E) Survival curves for patients who received chemotherapy and for those who did not (*P* = 0.046). (F) Survival curves for patients who received different radiotherapy doses (*P* = 0.014). (G) Survival curves for all 60 ATC patients who received surgery plus postoperative radiotherapy or another therapy (*P* = 0.002). (H) Survival curves for stage IVB patients who received surgery plus postoperative radiotherapy or surgery alone (*P* = 0.050).

The univariate analysis revealed that sex, primary tumor size, nodal status, and type of surgery were not significantly associated with prognosis. However, age, WBC count, PLT count, distant metastasis status, clinical tumor-node-metastasis stage, chemotherapy, radiotherapy, and therapeutic regimen significantly influenced the prognosis of patients with ATC (Table 1, Figure 1A-G). In the present study, surgery did not affect survival (Table 1), because the distant metastasis rate was lower in the group that underwent surgery (7/41, 17.1%) than in the nonsurgical group (9/19, 47.4%) (*P* = 0.014), it can be excluded that the failure of surgery to improve survival was related to distant metastasis. The patients who received chemotherapy had a worse prognosis than those who did not (Table 1). To control the influence of distant metastasis status, a stratified analysis was performed. Among the 16 patients with distant metastasis, the median duration without chemotherapy was shorter than with chemotherapy, although the difference was not significant (2 months versus 3 months, respectively; *P* = 0.859). However, among the 44 patients without distant metastasis, the median duration without chemotherapy was significantly longer than with chemotherapy (11 months versus 7.5 months; *P* = 0.038).

Among the 34 patients with upper airway obstruction, 5 patients who received tracheostomy had a worse prognosis (all died, 1 each at 2, 3, 4, 11, and 36 months after the initial diagnosis) than did those patients who did not receive tracheostomy (3- and 5-year OS rates were 23.0% and 16.3%, respectively), although the difference was not significant (χ^2^ = 0.484, *P* = 0.487). 

Forty patients with stage IVB disease were selected to investigate the effects of postoperative radiotherapy. Seventeen patients were excluded from the analysis: 10 did not undergo surgery, 3 underwent surgery plus postoperative chemotherapy, and 4 underwent surgery plus postoperative chemoradiotherapy. The remaining 23 patients were divided into a surgery group (n = 15) and a surgery plus postoperative radiotherapy group (n = 8) (radiotherapy dose range: 40–70 Gy). The patients in the latter group had a better prognosis (*P* = 0.050) (Table 2, Figure 1H).

**Table 2:** Effect of postoperative radiotherapy in 23 patients with AJCC stage IVB ATC.

Treatment	n	Deaths(n)	Postoperative residual tumor rate (%)^a^	OS rates (%)			*P*
				1-year	3-year		
S	15	14	13 (86.7)	20.0	10.0		0.050
S + X	8	7	7 (87.5)	62.5	37.5		

Abbreviations: AJCC, American Joint Committee on Cancer; ATC, anaplastic thyroid carcinoma; OS, overall survival; S, surgery; X, postoperative radiotherapy.

^a^ No significant difference in the rates of residual disease before radiotherapy between group S and group S + X (*P* = 0.956).

#### Multivariate analysis

For the multivariate analysis, the significant variables from univariate analysis including age, WBC count, distant metastasis, clinical tumor-node-metastasis stage, chemotherapy, radiotherapy, and therapeutic regimen were finally included in the Cox proportional hazards model that included all 60 patients. WBC counts and surgery plus postoperative radiotherapy (*P* < 0.05) were identified as independent prognostic factors in patients with ATC (Table 3). 

**Table 3:** Cox proportional hazards model of all 60 patients with ATC.

Factor	aHR*	95% CI	*P*
WBC count			
< 10.0 × 10^9^/L	1.000		
≥ 10.0 × 10^9^/L	1.869	1.069–3.269	0.028
Therapeutic regimens			
Other therapy	1.000		
S + X	0.392	0.200–0.770	0.006

Abbreviations: ATC, anaplastic thyroid carcinoma; HR, hazard ratio; CI, confidence interval; WBC, white blood cell; S, Surgery; X, postoperative radiotherapy.

* Adjusted by age, WBC count, distant metastasis, clinical tumor-node-metastasis stage, chemotherapy, radiotherapy, and therapeutic regimen.

### Expression of EPO, IL-6, IL-11, CSF1 gene by immunohistochemistry assay

In order to explore the correlations of expression of some growth factors with increase in WBC count and PLT count in the patients with ATC, we examined the expression of 4 genes (EPO, IL-6, IL-11, CSF1) in 22 patients who had tissue specimens available for analysis by IHC. We found that there were positive correlations between WBC count and CSF1 (r = 0.370, *P* = 0.045) as well as IL-11(r = 0.569, *P* = 0.003); but we did not find that either CSF1or IL-11 was significantly correlated with PLT count. Unfortunately, we did not find a significant correlations of either EPO or IL-6 expression with prognosis, WBC count, and PLT count in these 22 patients with ATC.

## Discussion

### Locoregional Treatment: Surgery and Postoperative Radiotherapy

Because most clinically apparent ATCs are unresectable at presentation, it is difficult to evaluate the benefits of surgical resection without introducing selection bias. Consequently, the significance of surgical resection of the primary tumor remains controversial [6].In the present study, the extent of resection did not affect survival (χ^2^ = 0.005, *P* = 0.998), which is similar to the findings of other studies [4,8,10,11]. However, most studies have found that curative resection significantly improved the survival of ATC patients [6,16–18]. Pierie et al[16] treated 44 of 67 patients surgically and reported 1- and 3-year OS rates of 92% and 83%, respectively, after the completed resections; 35% and 0%, respectively, after debulking; and 4% and 0%, respectively, after no resection. 

It is also difficult to determine surgical management of upper airway obstructions [19–23]. Patients with upper airway obstruction could be considered for treatment with tracheostomy [21]. However, not all patients with upper airway obstruction benefit from tracheostomy. Hölting et al [22,23] described 69 of 170 patients with locally infiltrating ATC who required a tracheostomy; in 45% of these 69 patients, a tracheostomy was performed as a prophylactic procedure; the follow-up data showed no benefit from this additional procedure. In the present study, among 34 patients with upper airway obstruction, the 5 patients who received tracheostomy had a worse prognosis than did those patients who did not receive tracheostomy, which supports the findings of Hölting et al [22,23]. In general, managing the airways of ATC patients continues to be a major challenge for physicians [20]. 

When used alone to treat ATC, surgery, radiotherapy, or chemotherapy rarely affects OS rates significantly [1].Therefore, multimodal therapy has increasingly become the treatment of choice for ATC. Because ATC is usually advanced at diagnosis and often cannot be resected completely, postoperative radiotherapy is desirable. Goutsouliak et al [12]reported that the median OS duration of 33 ATC patients who received doses < 40 Gy was significantly shorter than that of 24 patients who received ≥ 40 Gy (3 versus 9 months, *P* < 0.05). Results published by Swaak-Kragten et al [24] or by Sherman et al [9] suggested the total radiation dose as an important prognostic factor. Similarly, in this study, the patients who received conventional radiotherapy with doses ≥ 40 Gy had a better prognosis than did those who did not undergo radiotherapy or who received doses < 40 Gy; Furthermore, we found that postoperative radiotherapy significantly improved the prognosis of the patients with stage IVB disease (Table 2, Figure 1H). Chen et al [18] also noted that the addition of radiotherapy to surgery improved survival in patients with disease extending into the adjacent tissue, whereas the patients with disease confined to the thyroid capsule or who had further extension or distant metastatic disease did not benefit from this approach. In addition to postoperative radiotherapy, Segerhammar et al reported that hyperfractionated radiotherapy that was administered pre-operatively may improve the locoregional control [8].

### Adjuvant Therapy: Chemotherapy

Because most ATC patients have advanced local disease or distant metastasis at presentation, most of them require chemotherapy. Doxorubicin alone or in combination with cisplatin is a common regimen, but the response rate with this regimen is low [6,7,25,26]. In recent years, many cytotoxic agents or regimens have been used to treat ATC; however, their effects remain disappointing [6,27,28]. The results in the present study indicate that chemotherapy did not benefit the patients without distant metastasis and may even have delayed opportunities for other treatments, which could increase survival. This result was different from the finding reported by Siironen [29]. They found that there were 44 patients with ATC (66% patients had a distant metastasis disease), and the patients who received chemotherapy (14 cases) had a significant longer median survival than that of the other patients (30 cases) who did not receive chemotherapy (8.5 versus 1.3 months, respectively) (*P* = 0.002). Unfortunately they did not provide details on the status of distant metastasis for the patients who received chemotherapy or not. Thus, the effect of chemotherapy on prognosis of the patients without distant metastasis is still needed for further investigation.

### Prognosis and Influencing Factors

In our study, patients 55 years or older at presentation had worse 1- and 3-year OS rates than did those younger than 55 years (*P* = 0.033). Our results support the findings of Kebebew et al [13], who conducted a study using the United States National Cancer Institute’s Surveillance, Epidemiology, and End Results database. Their cohort comprised 516 patients with ATC. The authors concluded that an age younger than 60 years and the combination of surgery and radiotherapy were independent predictors of lower cause-specific mortality rates. Age was proved to be a negative prognostic factor for the patients with ATC in most studies [5,9,13,16,30,31]. We also found that patients with WBC counts ≥ 10.0×10^9^/L had worse outcomes, which supports the findings of other studies [5,14,28]. A multivariate analysis showed that the WBC count was an independent prognostic factor, with a hazard ratio of 1.869. WBC counts are elevated in ATC in part because some ATC cells produce several growth factors and cytokines, such as colony-stimulating factor (CSF), interleukin-1α (IL-1α), IL-3, and IL-6, which lead to leukocytosis [32–34]. Using *Csf1* ^*op/op*^ mice, which are deficient in the macrophage growth factor CSF1, a recent study [35] has demonstrated a crucial role for macrophages in regulating tumor progression. Because CSF can also stimulate PLT growth, we analyzed our patients’ PLT counts from their medical records. The PLT count was measured for 42 patients at their initial evaluations; the 30 patients with PLT counts < 300.0 × 10^9^/L had better 1- and 3-year OS rates than did those with counts ≥ 300.0 × 10^9^/L (Table 1, Figure 1C). To our knowledge, this is the first report of a relationship between the peripheral PLT count in ATC patients and patient outcomes. Unfortunately, eighteen patients did not receive the PLT count at presentation, and this variable was not included in the multivariate analysis. In the present study, although we found that a positive correlation between the expression of CSF1 or IL-11 and WBC count but not for PLT count, these results, which could be biased by our small patient’s number, need to be further validated in future larger study.

Because of its aggressive nature, ATC is classified as AJCC stage IV, regardless of tumor size, nodal status, or distant metastasis status [15]. In the present study, primary tumor size was not related to the prognosis of ATC patients, although other authors have reported that large tumors correlate with worse outcomes [2,5,8,14,18,36]. As in most reports [10,14,28], our study did not find lymph node metastasis (*P* = 0.230) to be a prognostic factor. However, the patients with distant metastasis had worse 1- and 3-year OS rates than did the patients without distant metastasis (*P* = 0.032), similar to the findings of other studies [5,8,13,14,18,28,29].

In the present study, we found that the patients who underwent surgery plus postoperative radiotherapy had the best prognosis of all treatment regimens (*P* = 0.002); this strategy was an independent prognostic factor with a hazard ratio of 0.392 (*P* = 0.006) in the multivariate analysis.

Sugitani et al reported a larger analysis with 677 patients with ATC, who were collected from 38 registered institutions in Japan over 13 years [5]. They found that the clinical varieties of ATC could be the prognostic factors, and the incidental type was significantly better than the other ATC types. The survival for anaplastic transformation at the neck lymph node(s) was better than that for the common type, and the anaplastic change at a distant site had the worst prognosis (*P* < 0.01). In the present study, our results did not support such a conclusion (*P* = 0.559). We speculate that our results could be a chance finding as our study sample size was small. They also found that older patients (age ≥ 70 years), presence of acute symptoms, WBC count ≥10.0 × 10^9^/L, large tumor, T4b tumor, distant metastasis were significant prognostic factors for poor survival, but unfortunately they did not evaluate the correlation between the PLT count and the prognosis of the patients with ATC.

We acknowledge that there are some limitations regarding the present study due to its retrospective nature. First, it was very unfortunate that 18 patients did not receive PLT counts examination at presentation which might decrease the strength of analysis. Secondly, the recruited patients underwent various treatment regimens, for example, different chemotherapy regimens were used in different period. Thirdly, evaluation of the complete resection could not be performed because of the absence of partial resection margin data. In spite of the above limitations, we found several valuable prognostic factors of ATC patients in our study, especially, to our knowledge, this is the first report demonstrating the elevated peripheral PLT count may be an adverse prognostic factor of ATC patients. 

In conclusion, we found the interesting relationship between the peripheral PLT count and survival of ATC patients for the first time. The prognosis for ATC is particularly poor for patients with distant metastasis, a WBC count ≥ 10.0 × 10^9^/L, a PLT count ≥ 300.0 × 10^9^/L, or an age ≥ 55 years. Accurate staging is crucial for appropriate treatment. For patients who have few risk factors and stage IVA/B disease, intensive treatment combining surgery with postoperative radiotherapy is recommended. To improve survival, particularly in patients with stage IVC ATC, innovative treatment strategies and multicenter prospective studies are needed.
